# The trend of incidence and burden of neurological disease in Iran between 1990 and 2017: Based on global burden of disease estimations

**Published:** 2019-07-06

**Authors:** Hedayat Abbastabar, Sama Bitarafan, Mohammad Hossein Harirchian

**Affiliations:** 1 Advanced Diagnostic and Interventional Radiology Research Center, Tehran University of Medical Sciences, Tehran, Iran; 2 Iranian Center of Neurological Research, Neuroscience Institute, Tehran University of Medical Sciences, Tehran, Iran

**Keywords:** Incidence, Global Burden of Disease, Disability-Adjusted Life Year, Neurological Disorders, Iran

## Abstract

Neurological disease contributes significantly to morbidity and mortality in different ages and geographic areas around the world. The purpose of the current study was to investigate the incidence and disability-adjusted life years (DALYs) trend of neurological disease in Iran during 27 years ago. We used the data of the Global Burden of Disease (GBD) Study to estimate the incidence and DALYs of neurological disease in Iran in different age groups between 1990 and 2017. Age groups were defined in 5 groups including < 5 years, 5-14 years, 15-49 years, 50-69 years, and ≥ 70 years. The incidence number of neurological disease during 1990 to 2017 increased from 7.5 million to more than 12 million and the incidence rate grew as much as 1400 per 100000 populations in Iran. Totally, headache, epilepsy, and Alzheimer were the most common neurological diseases according to incidence and had the most values of DALY in Iran. The highest incidence and DALY of neurological disease was observed in the age group of 15-49 years. This study showed that the incidence and burden of neurological diseases had a dramatic increasing trend during 27 years ago in Iran. Consequently, it is necessary to investigate the causes of the growing trend in future studies.

## Introduction

One of the responsibilities of the World Health Organization (WHO) is to focus on research in areas that are important for public health promotion. Recently, WHO has been particularly interested in frequent and disabling neurological disorders with high burden on the world.   ^1^^-^^3^ Neurological disorders occur among all age groups and different geographical regions. They significantly contribute to morbidity and mortality around the world. ^4^^,^^5^ 

Increased life span and decreased fertility have caused a demographical transmission from mainly young populations to older and ageing ones, causing increases in the cognitive disorders such as Alzheimer disease (AD) and other dementias and Parkinson’s disease (PD).^   6 ^ In addition, neurological disorders cause a lot of costs to patients and the treatment system in the countries. A research accomplished in Europe during 2004 calculated that the yearly expenditure of neurological disorders [such as dementia, epilepsy, migraine and other headaches, multiple sclerosis (MS), PD, and stroke] reached €139 billion (nearly US$180 billion). The mentioned research only counted direct non-medical expenditures (e.g., society surveillance and informal care) and dismissed indirect charges and intangible expenditures.^       7^

Currently, it is estimated that neurological diseases and their squeals affect as many as a billion individuals in the world. They affect hundreds of millions of people in the world: more than 50 million people have epilepsy and about 47 million people are affected by dementia globally - AD is the most common cause of dementia and may contribute to 60%-70% of cases. The prevalence of migraine is more than 10% worldwide. ^8^^,^^9^

Since the neurological diseases often produce long-dated disability and most of them occur at an early age, prevalence and mortality indices underestimate their disability in the population. Therefore, it is necessary to be a combined measure of survival and health status among survivors to create a single measure. ^9^^,^^10^ To determine the burden of disease, the 1990 Global Burden of Disease (GBD) Study introduced a time-based measure that considers both premature mortality [years of life lost (YLL) due to premature mortality] and disability [years of healthy life lost due to disability (YLD)]. The summation of these two parts provides disability-adjusted life years (DALYs) - an indicator of the latter path of healthy life (years expected to live in full health status) lost due to the occurrence of specific diseases and injuries. One DALY can be considered as one of the lost healthy life years and the burden of disease as an index of the difference between present health status and ideal health condition in which anyone lives in old age without disease and disability.^   11 ^ Epidemiological study plays a main role in the identification of disease occurrence and patterns as well as related risk factors and etiology.^   12 ^ The purpose of the current study was to investigate the incidence and DALY trend of neurological disorders in Iran during the years of 1990 and 2017.

## Materials and Methods

***Study type:*** Using data from the GBD study of 1990 to 2017, this study presented the trend of incidence and DALYs of neurological disorders in Iran by age groups. The 2017 GBD study was a comprehensive and systematic effort to calculate the global and regional comparative risk assessment of morbidity, mortality, and DALYs caused by different risk factors and diseases according to data-gathering and estimations of 354 types of diseases and injuries and 282 causes of death in 195 countries.^   13 ^

***Data source:*** The process for non-fatal evaluation begins with the incorporation of data sources from several possible origins which include 21 possible Global Health Data Exchange (GHDx) data types ranging from scientific literature to epidemiological surveillance data. GBD collaborators network provided 2842 data sources for GBD 2017. They analyzed 21100 sources of epidemiological surveillance data (country-years of disease reporting) for GBD 2017 and 4734 sources of disease registry data. For non-fatal estimation, they did systematic data and literature searches for 82 non-fatal causes and one impairment, which were updated in February 11, 2017.^   13 ^

Calculations were performed for age groups of < 5, 5-14, 15-49, 50-69, ≥ 70 years as well as all age groups. AD, PD, epilepsy, MS, motor neuron diseases (MNDs), other neurological disorders, and all neurological diseases were included in the study. Firstly, through a comprehensive systematic review of published and unpublished data, a Bayesian meta-regression method was performed to ensure consistency between incidence rate and cause of death for each condition.^   14 ^ In this study, each measure of incidence and DALY were presented in three ways: number, rate, and percentage. The associated number of each event in each age group was divided into the population in groups and expressed in 100000 populations for the rate calculation. In addition, the number of related cases in each age group was divided into the total number of that event and multiplied in 100 for estimation of proportion. All calculations and statistical analysis were performed by GBD Results Tool software.

## Results

***Incidence of neurological disorders***

*Numbers:* According to GBD, the number of total neurological disorders new cases in all age groups, during the years of 1990 and 2017, significantly increased from about 7.5 million to more than 1200 million. During the same period, the incidence numbers of each neurological disease increased in all age groups and most of the growth was related to headaches, AD, and epilepsy, respectively. Also, based on the relationship of incidence cases of neurological disorders with age, except for epilepsy and MNDs, the number of other diseases was ascending and raised with age and the most number of all neurological disorders were seen in the age group of 15-49 years (Table 1, A). 

*Rates:* According to GBD, during the years of 1990 and 2017, the incidence rate of all neurological disorders increased as much as 1400 per 100000 populations in all age groups. According to the type of disorders, the highest values were related to headaches, epilepsy, and AD, respectively, both in 1990 and 2017; but AD surpassed epilepsy in 2017. Moreover, in terms of the relationship of neurological disorders with age, epilepsy mostly occurred in age group of 5-14 years; headache, MNDs, and MS were more prevalent in 15-49 years age group, and the rest disorders were more seen in the age group of 70 and more years old (Table 1, B).

*Proportion:* According to GBD, both in 1990 and in 2017, from 100% incidence cases of neurological diseases, more than 99% were due to headaches followed by AD and epilepsy, respectively. Moreover, by comparing 1990 and 2017, except for headaches, the incidence proportion of most diseases increased in any age group; the most increases were seen for AD (0.27%), epilepsy (0.06%), and PD (0.04%). In addition, more than 55% of all neurological disorders firstly occurred in the age group of 15-49 years in 1990; also, this age group had the highest proportion (66% of all neurological disorders incidence) in 2017 (Table 1, C).

***DALYs of neurological disorders***

*Numbers:* According to GBD, during years of 1990 and 2017, the number of all neurological disorders DALY nearly doubled in all age groups. In addition, the DALY number of most disorders increased during these years in all age groups and the highest values corresponded to headaches, AD, and PD, respectively; in contrast, the epilepsy burden decreased during these years. It is interesting to note that both in 1990 and 2017, more than two thirds of burden of all neurological disorders were seen in the age group of 15-49 years (Table 2, A).

*Rates:* According to GBD, although the burden of all neurologic disorders increased during 1990 to 2017 in all age groups, its value was halved in the first age group and slightly decreased in the second age group. It appears that except for epilepsy, the DALY rate of other diseases increased during these years in all age groups and the most growth was related to headaches, AD, and PD, respectively (Table 2, B).

*Proportion:* According to GBD, headaches, epilepsy, and AD had the most significant role in the burden of diseases during years of 1990 and 2017. In 2017 compared with 1990, the DALY percentage of AD, PD, and MS increased in all age groups, but the corresponding values of epilepsy and headache declined. The highest DALY of all neurological disorders occurred in the group of 15-49 years both in 1990 and 2017, but the age distribution of each disease was different from other ones (Table 2, C).

**Figure 1:**
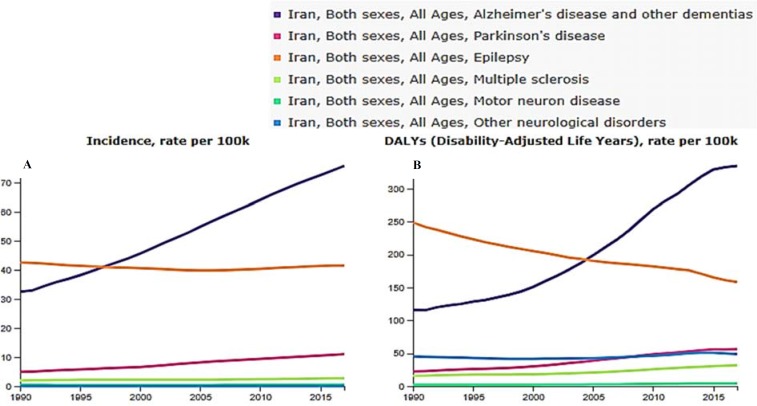
All ages' incidence (A) and disability-adjusted life years (DALYs) (B) rate of neurological disorders per 100000 populations in Iran from 1990 to 2017

In figure 1, all ages incidence (part A) and DALY (part B) trend of most important neurological disorders such as AD and PD were presented without considering age structure changes of population since 1990 to 2017. Approximately, the movement direction of the incidence and burden of all diseases is similar. Interestingly, during this time, the incidence and burden of AD increased significantly; in contrast, epilepsy figures had a good downward trend; but there was no significant change in the statistics of other neurological diseases.

**Figure 2:**
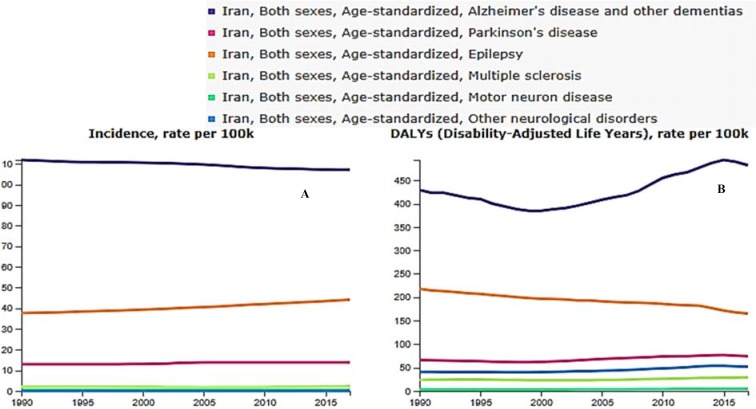
Age-standardized incidence (A) and disability-adjusted life years (DALYs) (B) rate of neurological disorders per 100000 populations in Iran from 1990 to 2017

Figure 2 shows the age-adjusted incidence (A) and DALY (B) trend of aforementioned neurological disorders from 1990 to 2017. By comparing it with figure 1, although it can be seen that AD incidence and burden and so epilepsy are still much higher than the rest of the disorders, their trend did not change significantly over time. On the contrary, the incidence of epilepsy increased during this time, and AD burden declined from 1990 to 2000.

## Discussion

The present study is a representative and population-based epidemiological study that was conducted on neurological disorders using GBD data in Iran.

**Table 1:** Distribution of neurological disorders incidence according to age groups during years of 1990 and 2017 in Iran as number, rate, and proportion

**Neurological disorders**	**Incidence by age groups in 1990**						**Incidence by age groups in 2017**					
	**< 5 **	**5-14**	**15-49**	**50-69**	**≥ 70**	**All ages**	**< 5**	**5-14**	**15-49**	**50-69**	**≥ 70**	**All ages**
**A: Number**												
AD	0	0	273	6347	12209	18829	0	0	777	13053	48468	62298
PD	0	0	3	1336	1202	2541	0	0	1025	3214	4825	9064
Epilepsy	7407	7887	293	1278	338	17203	7296	7010	14946	3446	1393	34091
MS	0	82	1008	28	4	1122	0	64	2115	78	12	2269
MND	137	12	33	57	16	255	113	9	91	155	62	430
Headache disorders	0	2381836	4396328	786304	128953	7693421	0	1744506	7989505	1868163	433137	12035311
Other neurological disorders	0	0	0	0	0	0	0	0	0	0	0	0
All neurological disorders	7545	2389618	4396328	795352	142722	7731565	7410	1751590	8008460	1888112	487900	12143472
**B: Rate per 100000**	**< 5**	**5-14**	**15-49**	**50-69**	**≥ 70**	**All ages**	**< 5**	**5-14**	**15-49**	**50-69**	**≥ 70**	**All ages**
AD	0	0	1.03	121.71	1259.90	32.54	0	0	1.65	107.48	1488.23	75.81
PD	0	0	1.11	25.62	124.05	4.89	0	0	2.17	26.46	148.15	11.03
Epilepsy	86.17	47.27	29.24	24.52	34.92	42.56	107.14	55.10	31.64	28.38	42.80	41.49
MS	0	0.49	3.82	0.54	0.38	1.94	0	0.51	4.48	0.65	0.38	2.76
MND	1.60	0.07	0.12	1.11	1.64	0.44	1.67	0.07	0.19	1.28	1.93	0.53
Headache disorders	0	14272.07	16653.97	15077.19	13307.33	13294.80	0	13712.59	16912.10	15381.00	13299.55	14645.76
Other neurological disorders	0	0	0	0	0	0	0	0	0	0	0	0
All neurological disorders	87.77	14319.90	16689.29	15250.69	14728.23	13377.18	108.81	13768.27	16952.22	15545.25	14981.04	14777.38
**C: Proportion**	**< 5**	**5-14**	**15-49**	**50-69**	**≥ 70**	**All ages**	**< 5**	**5-14**	**15-49**	**50-69**	**≥ 70**	**All ages**
AD	0	0	1.45	33.71	64.84	0.24	0	0	1.25	20.95	77.80	0.51
PD	0	0	0.12	52.58	47.30	0.03	0	0	11.31	35.46	53.23	0.07
Epilepsy	43.06	45.85	1.70	7.43	1.96	0.22	21.40	20.56	43.84	10.11	4.09	0.28
MS	0	7.31	89.84	2.50	0.36	0.01	0	2.82	93.21	3.44	0.53	0.02
MND	53.73	4.71	12.94	22.35	6.27	0	26.28	2.09	21.16	36.05	14.42	0
Headache disorders	0	30.96	57.14	10.22	1.68	99.51	0	14.49	66.38	15.52	3.60	99.11
Other neurological disorders	6.50	0	0	0	0	2.13	9.05	0	0	0	0	0.50
All neurological disorders	0.10	30.90	56.87	10.28	1.85	100	0.06	14.42	65.95	15.55	4.02	100

AD: Alzheimer's disease; PD: Parkinson’s disease; MS: Multiple sclerosis; MND: Motor neuron disease

**Table 2:** Distribution of neurological disorders disability-adjusted life years (DALYs) according to age groups during years of 1990 and 2017 in Iran as number, rate and proportion

**Neurological disorders**	**DALY by age groups in 1990**						**DALY by age groups in 2017**					
**A: Number**	**< 5**	**5-14**	**15-49**	**50-69**	**≥ 70**	**All ages**	**< 5**	**5-14**	**15-49**	**50-69**	**≥ 70**	**All ages**
AD	0	0	495	19391	47421	67117	0	0	1358	42320	231425	275103
PD	0	0	720	5503	6779	13003	0	0	2098	12998	31107	46204
Epilepsy	50549	40432	44555	6788	1784	144109	16840	24901	67364	14917	6163	130186
MS	0	61	6277	2410	348	9097	0	48	17122	7481	1531	26183
MND	196	56	290	486	46	1076	148	47	1028	1929	255	3407
Headache disorders	0	66556	448167	74433	7921	597079	0	51899	849969	182199	25400	1109467
Other neurological disorders	8559	7306	8106	1901	311	26148	5935	7677	18713	6259	1523	40109
All neurological disorders	59305	114412	508612	110915	64421	857667	22924	84572	957653	268105	297406	1630661
**B: Rate per 100000**	**< 5**	**5-14**	**15-49**	**50-69**	**≥ 70**	**All ages**	**< 5**	**5-14**	**15-49**	**50-69**	**≥ 70**	**All ages**
AD	0	0	1.88	371.82	4837.97	115.99	0	0	2.87	348.43	7105.94	344.77
PD	0	0	2.73	105.52	699.65	22.47	0	0	4.44	107.02	955.16	56.23
Epilepsy	588.01	242.29	168.78	130.16	184.12	249.04	247.28	195.73	142.60	122.82	189.25	158.42
MS	0	0.37	23.78	46.23	35.90	15.72	0	0.38	36.24	61.60	47.03	31.86
MND	2.29	0.34	1.10	9.32	4.79	1.86	2.18	0.37	2.18	15.88	7.83	4.15
Headache disorders	0	398.84	1,697.73	1427.25	817.45	1031.82	0	407.95	1799.21	1500.02	799.91	1350.11
Other neurological disorders	99.57	43.78	30.71	36.45	32.13	45.25	87.16	60.35	39.61	51.54	46.77	48.81
All neurological disorders	689.87	685.62	1926.70	2126.77	6648.02	1482.15	336.61	664.77	2027.15	2207.28	9131.90	1984.35
**C: Proportion**	**< 5**	**5-14**	**15-49**	**50-69**	**≥ 70**	**All ages**	**< 5**	**5-14**	**15-49**	**50-69**	**≥ 70**	**All ages**
AD	0	0	0.74	28.89	70.65	7.83	0	0	0.49	15.38	84.12	16.87
PD	0	0	5.54	42.32	52.13	1.52	0	0	4.54	28.13	67.33	2.83
Epilepsy	35.08	28.06	30.92	4.71	1.24	16.80	12.94	19.13	51.74	11.46	4.73	7.98
MS	0	0.67	69.00	26.49	3.83	1.06	0	0.18	65.39	28.57	5.85	1.61
MND	18.22	5.20	26.95	45.17	4.28	0.13	4.34	1.38	30.17	56.62	7.48	0.21
Headache disorders	0	11.15	75.06	12.47	1.33	69.62	0.00	4.68	76.61	16.42	2.29	68.04
Other neurological disorders	32.73	27.94	31.00	7.27	1.19	3.05	14.80	19.14	46.66	15.60	3.80	2.46
All neurological disorders	45.75	7.44	34.78	7.58	4.47	100	11.17	4	53.01	14.78	16.70	100

DALY: Disability-adjusted life year; AD: Alzheimer's disease; PD: Parkinson’s disease; MS: Multiple sclerosis; MND: Motor neuron disease

Epidemiological studies identify the risk factors and pattern of disease. According to the current study, both in 1990 and 2017, headaches, epilepsy, and AD were the most common neurological diseases, respectively. During these years, incidence and DALY of total neurological disorders impressively increased in all age groups. The highest incidence and DALY numbers and percentages of all neurological disorders were seen in the 15-49-year group. 

According to GBD, during the years 1990 and 2017, the total number of neurological disorders increased from about 7.5 million to more than 12 million, the incidence rate was over 1400 per 100000 populations, the DALY number almost doubled, and the DALY rate increased in all age groups. Neurological disorders consist 2% of the GBD, in the event that cerebrovascular disease (CVD) and some of the neuroinfections [poliomyelitis, tetanus, meningitis, and Japanese encephalitis (JE)] accounted for 4.3% of the GBD in 2005. As a result, neurological disorders account for 6.3% of the GBD. 12 Neurological disorders contribute to 92 million DALYs in 2005 projected to increase to 103 million in 2030 (approximately 12% increase).^   12 ^ In a study conducted by the Neurological Disorders Collaborator Group, neurological disorders with 10.2% of global DALYs and 16.8% of global deaths were considered as the major cause of DALYs and the second largest cause of death in 2015. Based on the GBD study between 1990 and 2015, the number of deaths due to neurological disorders increased by 36.7% and the number of DALYs increased by 7.4%. However, in the GBD study, in addition to our study, neurological disorders, stroke, meningitis, encephalitis, tetanus, and nervous system cancers were also included.^   15 ^ The results of most studies indicate that the incidence and burden of neurological diseases increased over time.   ^16^^-^^19^ The reasons should be investigated; because it may be partly due to the aging population, the improvement of therapeutic facilities and more patient survival, or the increased exposure to hazardous exposures associated with neurological diseases. ^20^^,^^21^ 

According to GBD, headaches, epilepsy, and AD were the most common neurological diseases according to incidence and so, they have the most significant role in the burden of disease in terms of DALY during 1990 and 2017 in Iran. Headaches are the most common human diseases. Migraine and severe headaches afflict more than 72 million Americans (22.7% of total population). In addition, the National Health and Nutrition Examination Survey (NHANES) estimates 16.2% of adults with migraine alone.^22^^,^^23^ AD is one of the prevalent neurological diseases in the United States, as it generally reaches 5.3 million, and 2.2 million people have other types of dementia. In total, it accounts for 7.5 million or 2.4% of the total population. ^24^^,^^25^ Epilepsy affects about 2.8 million Americans, nearly 1.0% of the total population, which affects more elderly people including black people and especially black men. Notwithstanding its high prevalence, the economic burden of epilepsy is lower than other neurological diseases. ^26^^,^^27^ In a systematic review conducted on neurological disorders, regional or geographical differences in their epidemiology were shown. For example, in this study, the highest incidence rate of epilepsy was seen in Africa (215 per 100000 persons/year) and the lowest was seen in Europe (42 per 100000 persons/year).^   28 ^ These variations are due to differences in the geographical distribution of risk factors as well as age and gender structure of the population.

According to GBD, the highest incidence and DALY numbers and percentages of all neurological disorders in both years of 1990 and 2017 were seen in the age group of 15-49 years; but the most significant rates of these measures were observed in the last age group (≥ 70 years). The reason for this difference is explained by the dependence of the number and percentage to the count of people who lived in each age group. That is, the number of people who live in the age group of 15-49 years is larger than the other groups; therefore, its number and percentage is higher.

However, the incidence rate is independent of the age group population and shows the risk of morbidity in different age groups. In a cross-sectional study conducted on elderly people with cognitive complaints (> 60 years) in the city of Kolkata, India, despite the limited age range of participants, the prevalence of neurological diseases increased in both women and men along with age.^   29 ^ The prevalence of many neurological diseases, especially neurogenic disorders, increases with age. For example, the meta-analysis of global data on PD showed the prevalence of 40.51 per 100000 people in the age group of 40-49, 106.67 per 100000 in people aged 50 to 59, 428.48 per 100000 in subjects aged 60-69, 1086.54 per 100000 people aged 70-79, and 1902.98 per 100000 people over the age of 80 years.^   30 ^

According to GBD, age-adjusted incidence and DALY from 1990 to 2017 showed that epilepsy incidence increased during this time, but the burden of AD decreased between 1990 and 2000 and then it had a rising trend. Increasing trend of occurrence and DALY of neurological disorders that has been observed in many studies, probably is due to the aging of population, because physiological aging begins after sixty years. Growth in both animal and human models is associated with changes in the performance of central cholinergic neurons. These alterations mainly involve reducing the level of cholinergic receptors, decreasing the synthesis and release of acetylcholine, and a significant reduction in the number of muscarinic cholinergic neurons that may be related to age-related memory impairment such as AD. ^30^^,^^31^ Nevertheless, it is recommended that in the future more studies examine the role of other factors in increasing the incidence and burden of neurological disorders.

Although GBD uses various data sources from scientific literature to epidemiological surveillance information for information collection and estimation, according to the Iranian neurologists opinion, the GBD values of some neurological disorders, in particular, for MS are very different and lower than the reality and their observed trend in the last 27 years. As a result, the need to set up neurological disorders registry system in Iran is felt more than ever before, because it could help policy makers in accurate estimation and predicting or preparing facilities in the future.

## Conclusion

The results of the current study showed that the incidence and burden of neurological disorders had a dramatic upsurge trend during the years 1990 and 2017 in Iran. Consequently, it is necessary to investigate the causes of this increase in future studies.

## Notes:

**How to cite this article:** Abbastabar H, Bitarafan S, Harirchian MH. The trend of incidence and burden of neurological disease in Iran between 1990 and 2017: Based on global burden of disease estimations. Iran J Neurol 2019; 18(3): 134-42.
